# Calprotectin in Cystic Fibrosis

**DOI:** 10.1186/1471-2431-14-133

**Published:** 2014-05-29

**Authors:** Nisreen Rumman, Mutaz Sultan, Khalil El-Chammas, Vi Goh, Nita Salzman, Diana Quintero, Steven Werlin

**Affiliations:** 1 Department of Pediatrics, Divisions of Pulmonary and Sleep Medicine, The Medical College of Wisconsin, Milwaukee, WI, USA; 2 Gastroenterology, The Children’s Hospital of Wisconsin, Milwaukee, WI, USA; 3 The current address: Department of Pediatrics, Makassed Hospital, Mount of Olives, P.O. Box 19482, Jerusalem, Israel; 4 The current address: Boston Medical Center One Boston Medical Center, Place Boston, MA 02118, USA; 5 The current address: Mercy Children’s Hospital and Clinics, Des Moines, IA 50314, USA

## Abstract

**Background:**

There is increasing evidence that intestinal inflammation plays a major role in gastrointestinal symptoms in cystic fibrosis (CF). Fecal calprotectin is a marker that is elevated in several gastrointestinal inflammatory diseases, but little is known about its value in CF. We aimed to look for associations of elevated fecal calprotectin among CF patients and whether its level correlates with the clinical manifestations of CF.

**Methods:**

A single stool specimen was collected from 62 patients with CF. Fecal calprotectin was measured using the commercially available ELISA kits (PhiCal^™^ test). Clinical data were collected from patients’ records and CF registry.

**Results:**

There were no significant differences between CF patients with normal and abnormal fecal calprotectin levels. However, patients who were not receiving inhaled antibiotics had higher fecal calprotectin levels than those who were.

**Conclusion:**

Elevated fecal calprotectin may not accurately predict intestinal inflammation in CF. However, the fact that it was elevated in both pancreatic sufficient and insufficient groups supports the concept of “cystic fibrosis enteropathy” regardless of the pancreatic status.

## Background

Cystic fibrosis (CF) is the most common cause of pancreatic insufficiency (PI) in children. Between 85% - 90% of CF patients have PI and malabsorption. These patients are typically treated with pancreatic enzyme replacement therapy (PERT). However, CF patients with pancreatic sufficiency (PS), and patients with PI who receive adequate PERT, may have persistent abdominal symptoms. It has been suggested that other poorly understood non pancreatic confounding factors are likely contributors [1].

There is no specific noninvasive test to prove the presence of intestinal inflammation. Several studies have shown evidence of intestinal inflammation in CF. Increased amounts of inflammatory markers were found in whole gut lavage indicating immune activation in the intestinal mucosa of CF patients [2-4]. A study using wireless capsule endoscopy (WCE) showed varying degrees of inflammatory findings including edema, mucosal breaks and ulcerations in most adult patients with CF [5].

Calprotectin, a protein found mainly in neutrophils, but also in monocytes and macrophages, is released during neutrophil activation or death. Calprotectin can be measured in plasma as well as other body fluids but is six times more concentrated in feces than in blood [6,7]. Fecal calprotectin (FC) is thus an inflammatory marker that is elevated in a variety of inflammatory intestinal diseases such as Crohn’s disease and CF [5,7,8]. Canani et al. showed a correlation between FC with the histologic grade of mucosal inflammation observed at endoscopy in patients with inflammatory bowel disease (IBD). In their study FC was a more accurate predictor of active mucosal inflammation than clinical scores and serum markers [8]. Similarly, Bruzesse et al. showed that intestinal mucosal inflammation is a major feature of cystic fibrosis. A comparison of 30 CF patients and 15 IBD patients with 30 healthy controls showed that the first 2 groups had significantly higher mean FC than controls [9].

The present study was performed to determine the frequency of elevated FC in CF patients, and to determine whether there are any associations with different characteristics such as pancreatic status, gastrointestinal morbidities, pulmonary function tests (PFTs) and pulmonary exacerbations. We also evaluated whether FC values differ between patients who receive certain treatments (PERT, probiotics and antibiotics) and those who do not.

## Methods

Between January 2009 – November 2010 all patients attending the CF clinic at the Children’s Hospital of Wisconsin were requested to provide a stool specimen for the study at their next clinic visit regardless of their age, CF mutations, pancreatic status or severity of disease. Sixty two patients participated. The samples were stored at (−60^0^C) until analysis. FC is stable at room temperature for up to 1 week [8,10,11].

Clinical data were collected from patients’ medical records and CF registry. Data collected included: age at CF diagnosis (years); age at sample collection (years); spirometry parameters including forced vital capacity (FVC), forced expiratory volume in 1 second (FEV1) and forced expiratory flow 25%-75% (FEF25-75%); gender; ethnicity; diagnosis (classic CF, or CF related metabolic syndrome - CRMS); body mass index (normal, obese, overweight); symptoms (abdominal pain, gas, vomiting, fever, pulmonary exacerbation); stool characteristics (frequency, consistency); hospitalizations; morbidities (meconium ileus, surgery, bowel resection, distal intestinal obstruction syndrome (DIOS); antibiotic use (inhaled, intravenous, oral); pancreatic enzyme supplementation; probiotic use; and pancreatic function (PI versus PS ).

### Calprotectin assay

The coded stool samples were thawed, aliquots of 80–120 mg were taken and then a quantitative measurement was done using commercially available ELISA kits (PhiCal^™^ test). ELISA steps were all completed according to the instructions of the manufacturer.

This study was approved by the institutional review board of the Children’s Hospital of Wisconsin and consent and/or assent was obtained from patients and/or parents as appropriate.

### Statistics

Descriptive statistics were used to summarize the sample’s characteristics. Chi square and Fisher’s exact test were used to assess demographic differences. Bivariate analysis was used to examine the effect of certain patient’s characteristics (use of inhaled, intravenous, or oral antibiotics), and FEV1 (normal being equal to or greater than 80% predicted) on FC levels. Analyses were performed for a normal FC level defined as less than 50 mcg/gm.

A statistical significance (alpha) level of 0.05 was used throughout, and SAS On Demand Enterprise Guide 4.2 (SAS Institute, Cary, NC) was used to perform all statistical analysis.

## Results

A total of 62 patients participated in the study. Three were excluded because of incomplete data. Data of 59 patients (33 females, 26 males) were analyzed. Forty three patients were PI and 16 patients were PS. Fifteen patients were < 6 years of age. In our clinic patients less than 6 years of age do not routinely perform PFTs. PFTs were available for 44 patients.

### Patient characteristics

The median patient age was 8 years. The median FC level was 94 mcg/gm (Table 1). There were no significant differences with respect to age at CF diagnosis, age at sample collection, and spirometry parameters between patients with normal and abnormal FC levels (Table 2). There was no statistically significant difference in FC levels between patients in regards to the other characteristics studied including gender, ethnicity, growth parameters, genotype, gastrointestinal symptoms (abdominal pain, distention/gas, vomiting, and stool pattern), being on PERT and/or probiotics, history of meconium ileus, bowel surgery/resection, history of distal intestinal obstruction syndrome (DIOS), number of pulmonary exacerbations and hospitalizations (Table 3). However, there was a significant statistical difference in regards to the use of inhaled antibiotics; those who were not on inhaled antibiotics had higher odds of having abnormal FC levels higher than 50 mcg/gm (*p* value 0.0101) (Table 4).

**Table 1:** Study population

**Factors**	**N**	**Median (range)**
Fecal calprotectin (mcg/gm)	59	94.29 (10–200)
Age at sample collection (yrs)	59	0.67 (0.04-4.58)
Age at diagnosis (yrs)	59	0 (0–2.17)
FVC (%)	44	100 (53–147)
FEV1 (%)	44	93.5 (25–139)
FEF25 (%)	44	85 (6–170)

**Table 2:** Pulmonary function test characteristics

**Factors**	**Normal**		**Abnormal**		**P-value**
	N	Median	N	Median	
Age at diagnosis (yrs)	18	0.04	41	0	**0.9718**
Age at sample (yrs)	18	6.00	41	9.00	**0.1956**
FVC (%)	15	105.00	29	99.00	**0.3040**
FEV1 (%)	15	106.00	29	92.00	**0.3040**
FEF25 (%)	15	92.00	29	84.00	**0.5858**

**Table 3:** Patient characteristics

**Factors**	**N**	**Normal FC (%)**	**Abnormal FC (%)**	**P-value**
**Gender**				**1.0000**
Female	33	30	70	
Male	26	31	69	
**Ethnicity**				**0.3778**
American Indian	1	100	0	
Black	2	0	100	
White	50	32	68	
Hispanic	2	50	50	
Mixed	1	0	100	
Other	3	0	100	
**Diagnosis**				**0.6639**
Classic CF (PI and PS)	52	29	71	
CRMS	7	43	57	
**Symptoms**				
Abdominal pain				**1.0000**
No	51	31	67	
Yes	8	25	75	
Gas				**0.2181**
No	56	29	71	
Yes	3	67	33	
Vomiting				**0.3027**
No	55	33	67	
Yes	4	0	100	
Fever				
No	59	31	69	
Yes	0	0	0	
**Exacerbation**				**0.7748**
No	37	32	68	
Yes	22	27	78	
**Stool frequency**				**0.6274**
1	16	31	69	
1 or 2	7	14	86	
1 or 3	1	0	100	
2	17	24	76	
2 or 3	5	40	60	
2 or 4	1	100	0	
3	4	25	75	
3 or 4	4	50	50	
4	2	50	50	
5 or 6	1	100	0	
7 or 8	1	0	100	
**Stool description**				**0.6394**
Formed	47	32	68	
Formed foul	1	0	100	
Formed greasy	1	0	100	
Foul greasy	1	100	0	
Loose	5	40	60	
Loose formed	2	0	100	
Loose foul	1	0	100	
Soft	1	0	100	
**Hospitalization**				**0.7187**
0	33	27	73	
1	9	33	67	
2	5	20	80	
3	7	57	43	
4	3	33	67	
7	1	0	100	
10	1	0	100	
**Meconium Ileus**				**0.7659**
No	40	33	68	
Yes	19	26	74	
**Surgery**				**1.0000**
No	40	30	70	
Yes	19	32	68	
**Intestinal resection**				**1.0000**
No	51	31	69	
Yes	8	25	75	
**Distal intestinal obstruction syndrome**				**1.0000**
No	58	31	70	
Yes	1	0	100	
**Inhaled antibiotics**				**0.0101**
No	35	17	83	
Yes	24	50	50	
**IV antibiotics**				**0.1604**
No	54	28	72	
Yes	5	60	40	
**Oral antibiotics**				**0.0880**
No	31	19	81	
Yes	28	43	57	
**Pancreatic enzyme**				**0.7533**
No	16	25	75	
Yes	43	33	67	
**Probiotic**				**0.0894**
No	57	28	72	
Yes	2	100	0	
**Pancreatic status**				**0.3433**
Insufficient	43	35	65	
Sufficient	16	19	81	

**Table 4:** Bivariate analysis of antibiotic use and fecal calprotectin (using normal cut off for fecal calprotectin of <50 Mcg/Gm)

		**%**	**OR**	**95% CI**	**P-value**
**Inhaled**					**0.0101**
	Normal	67	R		
	Abnormal	29	0.21	0.06, 0.68	
**IV**					**0.1604**
	Normal	17	R		
	Abnormal	5	0.26	0.04, 1.69	
**Oral**					**0.0880**
	Normal	67	R		
	Abnormal	39	0.32	0.1, 1.02	

### Bivariate analysis

Results of the bivariate analysis are summarized in Tables 4 and 5. The only significant finding was that patients with a normal FC level compared to those with levels above 50 mcg/gm, had higher odds of being on antibiotics (inhaled, intravenous, or oral).

**Table 5:** Bivariate analysis of FEV1 and fecal calprotectin (using normal cut off for fecal Calprotectin of <50 mcg/gm)

		**%**	**OR**	**95% CI**	**p-value**
**FEV1**					**0.4884**
	Normal	20	**R**		
	Abnormal	34	**2.11**	0.48, 9.24	

## Discussion

The evidence that factors other than PI and malabsorption play a role in “CF enteropathy” is increasing [2,5,9,12,13]. Intestinal inflammation may be one of the factors that result in persistence of GI symptoms even in the patients who are PS or PI patients receiving adequate PERT.

In the CF lung, there is still controversy whether inflammation precedes or follows infection. While Armstrong et al. found that inflammation follows respiratory infections [14,15], other studies of broncho-alveolar lavage fluid in infants with CF found elevated inflammatory markers early in the course of the disease even in the absence of bacterial colonization or infection [16,17]. This suggests that inflammation might be the earliest event in the CF lung causing damage and thus predisposing to infection, and that the basic defect in cystic fibrosis transmembrane regulator (CFTR) itself may initiate or amplify inflammation.

As with the lungs, the CFTR expression is high in the intestines. Dysregulation of the inflammatory response is thought to be present in tissues that express CFTR [2]. This could explain why PERT treatment does not completely correct gastrointestinal symptoms and why patients with PS may still have gastrointestinal symptoms. Bruzzese et al. showed that probiotics reduce intestinal inflammation and decrease pulmonary exacerbations and hospital admissions in patients with CF, this indicates that there may be a relationship between pulmonary and intestinal inflammation in CF [18].

Intestinal inflammation is a typical feature in CF [13]. This may be related to several other factors in addition to a dysregulated inflammatory response associated with the basic cellular defect of CFTR [2]. High doses of PERT can cause inflammation and fibrosing colonopathy [19]. Multiple factors predispose CF patients to small intestinal bacterial overgrowth (SIBO) which can cause inflammation, mucosal damage and maldigestion [1,18]. A local intestinal mucosal defect, believed to be present in CF patients, may explain the increased permeability to sugars and the disacchariduria that characterizes CF patients [20].

The gold standard for the detection of intestinal mucosal inflammation is endoscopy and biopsy, which are invasive and expensive. There is no other specific noninvasive test to accurately detect intestinal inflammation. But, based on previous studies correlating FC levels with the severity of inflammation in other inflammatory conditions, mainly inflammatory bowel disease (IBD), we looked for evidence of intestinal inflammation in CF patients by measuring FC [8,10,11].

FC in CF has been previously evaluated in 2 studies. Bruzzese et al. found that FC was elevated in 27 of 30 pediatric CF patients [9]. In 10 patients FC normalized after treatment with a probiotic, lactobacillus GG (LGG), suggesting the possibility of bacterial overgrowth. In the same study rectal nitric oxide (NO) production, another non invasive marker of intestinal inflammation that is elevated in the stool of children with active IBD was increased, thus supporting the FC results. NO production also decreased after LGG [9]. In the second study, FC was measured along with wireless capsule endoscopy (WCE) to quantify and localize intestinal inflammation in patients with CF and relate these findings to the clinical status and pancreatic phenotype [5]. The images showed generalized enteropathy unrelated to pancreatic function status. FC was measured in 30 patients. It was normal (<50 mcg/gm) in all 9 PS patients as well as in all patients in the control group, but elevated in 18/21 of the PI patients indicating intestinal inflammation only in the patients with PI [5].

We decided to identify the role of calprotectin in our CF population. In contrast to the described studies, we found that 10/16 PS patients had elevated FC levels comparable to those of PI patients. Since FC is a measure of inflammation, this indicates that intestinal inflammation is also present in PS patients and implies that inflammation is part of the disease process in CF regardless of the pancreatic status and is not related to PERT. In the present study there was no correlation between FC and any of the characteristics studied nor was there a difference in FC levels between PI and PS patients. Only 19 patients in our study (6/16 PS and 13/43 PI) had levels < 50 mcg/gm. This finding supports the concept of “CF enteropathy” as an independent entity in the disease process.

Pulmonary function tests were available for 44 patients. Patients with lower FEV1 were more likely to have higher FC levels; however this was not statistically significant. Lower FEV1 indicates worse pulmonary status, and hence probably more sputum production which if swallowed could contribute to increased intestinal inflammation. Excluding the sputum from the GI tract is impossible since the muco-ciliary escalator cannot be turned off in vivo [2,12]. Bacteria and other contents of swallowed sputum might be involved in direct stimulation of the intestinal mucosa. Calprotectin in the sputum itself might also increase the fecal levels. Golden et al. studied plasma calprotectin levels as a marker of pulmonary inflammation in CF. They found that plasma calprotectin was significantly higher in CF patients compared to matched controls [21]. Both sputum and serum calprotectin significantly decreased following treatment of a pulmonary exacerbation in a study by Gray et al. [22]. In our study, there was no significant difference in FC levels between patients who had a pulmonary exacerbation within one month prior to sample collection and those who had no recent exacerbations. The patients who were receiving inhaled antibiotics had significantly lower FC levels. But this was not true for oral or intravenous antibiotic usage (within one month prior to sample collection).

An altered intestinal microbiome may be a stimulus for inflammation and thus elevated FC levels. CF patients have multiple risk factors for small intestinal bacterial overgrowth (SIBO) including inspissated intestinal secretions, constipation, slow intestinal motility and frequent courses of antibiotics [23]. The frequent use of acid blockers, particularly proton pump inhibitors, in CF patients is another trigger for SIBO [23]. Lisowska et al. studied evidence of SIBO in 25 CF patients using the hydrogen-methane breath test, and at the same time measured the FC levels in those patients [24]. They found similar FC levels in both SIBO positive and negative patients, concluding that SIBO does not correlate with intestinal inflammation in CF. We did not assess our patients for SIBO.

We are aware that our study had several limitations: It is a small study at only one CF center, there was no control group and only one sample was obtained. Larger multicenter prospective studies may help determine if serial and longitudinal calprotectin levels may have clinical relevance during symptomatic episodes and whether certain interventions will have an impact on these levels. However, the finding of abnormal calprotectin levels in the pediatric population brings up the question about early detection of CF enteropathy. In addition, our observation of lower levels of calprotectin in individuals on inhaled steroids may prove that bacterial control in the intestinal tract may be also beneficial.

## Conclusion

There is increasing evidence that intestinal inflammation is part of the disease in CF patients. The causes of this inflammation may be multifactorial, and could be contributing to the gastrointestinal symptoms in PS patients and PI patients receiving apparently adequate PERT. FC is used as a marker of inflammation and although it may not be as an accurate indicator of intestinal inflammation in CF as it is in other inflammatory conditions of the intestines the fact that it was elevated in both PS and PI groups supports the concept of “CF enteropathy” regardless of the pancreatic status. This study highlights the limitation of an increasingly popular test as a marker of intestinal inflammation in the CF population. However, a larger cohort of patients is needed to confirm these findings.

## Competing interests

The authors declare that they have no competing interests.

## Authors’ contributions

NR participated in the design of the study, recruiting patients and collecting data, carried out the ELISA tests, and wrote the manuscript. MS participated in recruiting patients and collecting data, carried out the Elisa tests, and helped to draft the manuscript. KC and VG performed the statistical analysis and helped to draft the manuscript. NS participated in the design of study, coordinated and supervised the ELISA tests, and helped to draft the manuscript. DQ participated in the design of the study and helped to draft and edit the manuscript. SW conceived of the study, participated in its design and coordination, and helped to draft and edit the manuscript. All authors read and approved the final manuscript.

## Pre-publication history

The pre-publication history for this paper can be accessed here:

http://www.biomedcentral.com/1471-2431/14/133/prepub
